# Effect of Two Organic Production Strategies and Ageing Time on Textural Characteristics of Beef from the Retinta Breed

**DOI:** 10.3390/foods9101417

**Published:** 2020-10-07

**Authors:** Susana García-Torres, Adoración López-Gajardo, David Tejerina, Estrella Prior, María Cabeza de Vaca, Alberto Horcada

**Affiliations:** 1 Meat Quality Area, Centro de Investigaciones Científicas y Tecnológicas of Extremadura (CICYTEX-La Orden), Junta de Extremadura, Guadajira, 06187 Badajoz, Spain; dologa19@hotmail.com (A.L.-G.); tejerinabarrado@yahoo.es (D.T.); yeyiprior@hotmail.com (E.P.); merycv@hotmail.com (M.C.d.V.); 2 Departamento de Ciencias Agroforestales, Escuela Técnica Superior de Ingeniería Agronómica, Universidad de Sevilla, 41013 Sevilla, Spain; albertohi@us.es

**Keywords:** organic beef, ageing, tenderisation speed, meat quality, sarcomere

## Abstract

The primary aim of this paper is to determine the influence of two organic production systems, organic grass-fed (OG) and organic concentrate-fed (OC), vs. a conventional concentrate-fed (CC) system; the second aim is to determine the influence of the ageing period on the physical parameters and texture properties of beef from the Retinta breed. Muscle samples from *Longissimus thoracis* were stored at 2–4 ± 1 °C for 0, 7, 14, and 21 days for the purposes of ageing. Analyses of pH, water losses (drip loss and cooking loss), Warner-Bratzler shear force, texture profile analysis (TPA), and histological analysis of muscle fibre were carried out. The results revealed that organic meat experienced lower drip loss and higher cooking loss than conventional meat. Although the meat of organic grass-fed animals was tougher initially, it showed a higher tenderisation speed in the first ageing days than OC and CC meats. The sarcomere length increased during the ageing period, which showed a negative correlation to shear force. According to its texture characteristics, the Retinta meat produced in organic systems could be recommended by its quality.

## 1. Introduction

In recent years, organic production systems have gained increasing economic importance in the meat sector. This is due to the increasing commercial and social acceptance of these kinds of products, which is mainly associated to aspects relating to food safety, food quality, animal welfare, and environmental benefits [1].

Organic food consumers are willing to pay a premium for organic products, as they see themselves contributing to environmental sustainability and higher standards of animal welfare [2]. Furthermore, organic products are perceived as healthier, which is an item of added value, increasing consumers’ willingness to pay a premium for them [3]. Napolitano et al. [4] reported that consumers are influenced by the available information on organic production and that they value not only meat quality, but also the ethical considerations associated with organic farming. Furthermore, Lee and Yun [2] suggested that hedonic attitudes, perceptions of nutritional content, organic welfare, and sensory appeal attributes lead to a positive intention to purchase organic meat. However, Sirieix and Tagbata [5] showed that the ethical concept alone is not sufficient for organic consumers, who also demand information about the characteristics and quality of the product. These issues, together with consumers’ direct experience with organic beef, play a major role in the repurchase decision [6] and thus avoid the discord between the expected and experienced quality [7].

Previous studies have been focused on factors regarding organic production systems, health issues, animal welfare, and the marketing of organic products [8,9,10]. Research on the quality characteristics of organic food products has mainly focused on animal nutrition aspects and its impact on the quality of the products [9], as well as the effects of organic food on human health [10,11].

In the case of organic meat, several studies indicated a higher content of ω-3 fatty acids in organic meat [9] as well as a higher nutritional value and bioactive compounds content in organic beef [12], [13]. On the other hand, the physical–chemical quality aspects involved in the decision to buy and repurchase organic meat, such as colour, water content, and oxidative status as well as their conservation in different types of packaging [11,13,14], have been less studied. Despite these studies, it is necessary to increase research in order to improve the knowledge on the attributes of organic meat to strengthen the organic meat quality brand [15].

In this sense, the tenderness is the most valued quality attribute by consumers in beef [8,15]; consumers are often prepared to pay a higher price if the tenderness of the beef is guaranteed [15,16]. However, this is a complex parameter that results from the combination of several factors, such as animal rearing, intrinsic characteristics such as breed, gender, muscle structure, and connective tissue content, or the histological characteristics of muscle fibres, mainly sarcomere length and muscle fibre biochemistry [17]. The effect of the production system on the texture of beef has been studied by different authors, who described that the meat from calves reared in grazing production systems is tougher than animals reared under conventional feed-lot systems [18,19,20]. However, the literature about organic beef and production system effects on the texture properties of meat is scarcer [14,19]. The ageing process provides substantial improvements in meat tenderness and has been extensively studied [21,22,23,24]. This process involves complex changes in the muscle metabolism at the post-slaughter period, which can vary depending on animal breed, the metabolic status, and extrinsic conditions, such as the rearing system and stress prior to slaughter [24,25].

In Southwestern Europe, traditional cattle production is based on the extensive grazing of rustic breed calves, such as the Retinta or Avileña breeds on Mediterranean rangelands known as *Dehesas*. The *Dehesa* is a managed, agrosilvopastoral ecosystem whose soils are acidic, shallow, and easily eroded. Holm and cork oak form the most representative tree cover. The climate is semi-arid Mediterranean. The pasture that grows in these soil and climate conditions comprises many species, most of which are annuals. Cattle form the predominant livestock, with breeds of high rusticity that are able to thrive in this difficult environment. According to Caballero and Mata [26], this system is easily convertible to organic production by adjusting farm’s health and management practices to the specifications of the EU’s organic regulation [27]. Extensive conditions often involve periods of pasture shortage due to a dependence on climate conditions [28], which is a problem for producers. Although the term “organic” is often associated with free-range livestock [29], organic farmers have an alternative way to raise organic cattle without depending on the seasonal availability of grass [8] through the use of organic concentrate. Retinta is a rustic bovine breed that is well adapted to the *Dehesa* ecosystem, and it produces meat of high sensorial quality [30], with a lower intramuscular fat content [31] and a higher content of polyunsaturated fatty acids than other improved breeds [32]. However, in the meat industry market, where the meat yield of the carcass is a quality characteristic, carcasses of the Retinta breed are disadvantaged compared to improved breeds of cattle. Among the strategies to improve their value on the market, and considering their rustic character, organic production could give an added value to Retinta calves.

Within this framework, this paper attempts to raise knowledge about organic beef quality. The aim of this research is to analyse the effects of two types of organic production systems (grass-fed and organic concentrate-fed) by comparing both types with conventionally produced Retinta beef, and the effect of ageing period (0, 7, 14, and 21 days) on texture properties of beef from the Retinta breed.

## 2. Materials and Methods

### 2.1. Experimental Design and Animal Management

For the present study carried out in three experimental farms in southwestern Spain, seventy-five Retinta male calves were selected and reared with their mothers’ milk until weaning at 8–9 months of age. At weaning, animals were allocated to three experimental groups ensuring that the average weight of each group was similar and where the calves were maintained since weaning until slaughter, as follows: organic grass-fed animals (OG, *n* = 30), organic concentrate-fed animals (OC, *n* = 30), and conventional concentrate-fed animals (CC, *n* = 15).

Calves in the OG system were fattened in a local agro-silvo-pastoral system called *Dehesa* in the experimental farm owned by CICYTEX (Center for Scientific and Technological Research of Extremadura, Spain). The calves were free grazing fed (from weaning to slaughter) on natural pasture resources from *Dehesa*, mainly composed of raygrass (*Lolium perenne* and *Lolium rigidum*) and clover (*Trifolium repens*), and they also received organic concentrate in controlled feeders when grass pasture was scarce (approximately 20% of the total dry matter supplied) according to Organic European Regulations [27]. The animals had freedom of movement and free access to water in the natural resources and waterers. Calves in the OC system were reared in pens allowing 8 m^2^ per animal, according to regulations on organic production [27] at the Divino Salvador Coop. farm (Cádiz, Spain). After weaning, the animals were fed on a 40% organic concentrate and 60% organic forage—25% barley straw and 35% grass silage—(dry matter basis). The organic concentrate was composed of barley grain (36.2%); oat grain (24.5%); peas (16.6%); sunflower seed cake (19.6%); and minerals and vitamins (3.1%). The CC system animals were assigned to this research by the Diputación de Cádiz Agricultural Station (Cádiz, Spain). The calves were confined in pens allowing 4 m^2^ per animal and fed on conventional concentrate *ad libitum* (approximately 80% of total dry matter supplied) and barley straw without the possibility of grazing. The conventional concentrate was composed of maize grain (34.0%); barley grain (33.5%); corn gluten feed (17.1%); soybean meal 44 (8.4%); minerals and vitamins (3.9%); and palm oil (3.1%).

The experimental procedures to which the animals were subjected during the fattening phase were in compliance; in the case of conventional production, they were considered standard farming practices and exempted from the consideration of ethical and welfare aspects by the Animal Care and Ethics Committee (CAEC). In the case of organic productions, animals were complying with animal welfare and organic regulations [27]. For all animals, Council Regulation (EC) No. 1099/2009 [33], for the protection of animals at the time of slaughter, was also complied.

Animals were slaughtered in 2 years (15 calves each year from each group) at their commercial weight in the local market in licensed slaughterhouses, which complied with animal welfare and organic regulations [27].

### 2.2. Muscle Sampling and Ageing Process

At 24h post-mortem, the pH_24_ was measured on the *Longissimus thoracis* (LT) muscle at the 6th rib each of the left half carcass, and after the LT muscles were removed between the 5th and 10th rib. The *Longissimus thoracis* muscles were filleted from the cranial to caudal area, and eight steaks were obtained. The first four consecutive steaks (2 cm thick chops) were assigned to determine pH, drip loss, and histological analysis, one for each studied ageing time, and the next four steaks (3.5 cm thick chops) were assigned to texture study by post-mortem ageing time. The first steak was immediately analysed (T_0_), and the fifth one was vacuum-packed in a plastic bag polyethylene (O_2_ permeability, 9.3 mL O_2_/m^2^/24 h at 0 °C) and frozen at −20 °C for texture analysis. The steaks T_0_ were analysed at 24 h post-mortem.

For the ageing process, the six remaining steaks were preserved individually, overwrapped with transparent oxygen-permeable polyvinyl chloride film for 7 (T_7_), 14 (T_14_), and 21 (T_21_) days at 2–4 ± 1 °C in a refrigerator (mod. AN1002, Infrico S.L., Sevilla, Spain). After each ageing time, the samples for pH, drip loss, and histological study were analysed immediately, and the steaks for texture were vacuum-packed (under the above conditions) and frozen at −20 °C until analysis.

### 2.3. Physical–Chemical Analysis

The pH values were measured using a penetration electrode coupled with a temperature probe (Crison pH-meter mod. 507. Crison Instruments, Alella, Barcelona, Spain). The pH assessment of the ageing samples (T_7_, T_14_, and T_21_) were measured after each ageing period.

Water losses were measured as drip loss (DL) and cooking loss (CL). DL was determined according to the method proposed by Honikel and Hamm [34] by duplicates on 50 g fresh samples taken and placed in a container (meat juice collector, Sarstedt, Nümbrecht, Germany) and kept at 4 ± 1 °C during the described ageing times. The results were expressed as water loss g/100 g of muscle between T_0_ and T_7_, T_7_ and T_14_, and T_14_ and T_21_. The cooking loss method is described below, in the section about the instrumental texture.

The texture of meat was instrumentally evaluated on cooked samples. Previously, the samples were thawed in cold water (at room temperature) for 3 h before testing until reaching an internal temperature of 17–20 °C. For the cooking process, the raw samples were weighed and vacuum-packed in nylon/polyethylene bags and cooked by immersion in a water bath preheated at 80 °C, with controlled temperature until the steak reached an internal temperature of 75 °C [35]. Cooked samples were left to cool under tap water, to prevent further cooking, for 30 min and then chilled overnight at 4 °C. The difference in weight before and after cooking was used to calculate for the determination of cooking losses (CLs), and the results were expressed as water loss g/100 g of muscle.

For the texture analysis, each steak was cut transversally into two halves to be used in a Warner-Bratzler (WB) device and subject to texture profile analysis (TPA) with a compression device. For texture assessment, 1 cm^2^ strips were made from each cooked steak, with the muscle fibers parallel to the longitudinal axis of the sample [36]. All texture measurements were taken using a texturometer TA-XT 2i Texture Analyser of Aname (Stable Micro Systems Ltd., Surrey, UK), and they were carried out with the sample at room temperature (22 ± 2 °C). Instrumental determinations were repeated 8 times per sample, and results were data averaged.

For WB analysis, the samples were cut with a Warner-Bratzler blade (HDP/BS) in the perpendicular direction to the muscle fibers. Three parameters were measured: maximum shear force (kg/cm^2^), shear firmness (kg·s), and total work (kg/s).

On the other hand, the TPA test was conducted to evaluate the textural profile of the meat according to Tejerina et al. [37]. The cooked samples were cut into uniform cubes of approximately 1 cm^3^ and were axially compressed to 20% (TPA20) of their original height using a probe with a 20 mm diameter flat plunger (P/20) connected to a load cell of 25 kg at a test speed of 2 mm/s. The samples were compressed in two cycle sequences, according to the recommendations for analysing food texture provided by Bourne [38]. TPA20 was used to determine the contribution of myofibrillar structures, without the intervention of connective tissue, on meat texture. The textural parameters obtained from force–deformation curves [39] were as follows: hardness (kg/cm^2^) = maximum force required to compress the sample (peak force during the 1st compression cycle); springiness (cm) = height that the sample recovers during the time that elapses between the end of the 1st compression and the start of the 2nd; chewiness (kg∙cm∙s) = the work needed to chew a solid sample to a steady state of swallowing; and resilience (dimensionless) = how well the product regains its original height, as measured on the first withdrawal of the cylinder.

### 2.4. Histological Analysis of Muscle

Histological analyses (sarcomere length and cross-sectional area of fibre) of LT muscle were performed. Samples were taken directly from the centre of chops of the LT muscle collected and aged (for 0, 7, 14, and 21 days at 4 °C) and were placed in glutaraldehyde (2.5% *v*/*v* in phosphate buffer pH 6.5). The method used to determine sarcomere length was described by Torrescano et al. [40]. From each of the samples in glutaraldehyde, 4 bundles were removed under a magnifying glass and placed on glass slides, after which they were contrast-stained with haematoxylin and eosin. Sarcomere lengths were measured using an immersion objective (×100) under a phase contrast microscope (Nikon Eclipse 50i model) and Nis-Elements 3.10 computer image analysis software. The result was the average of 150 measured lengths (μm). Serial cross-sections were obtained according to described method by Abreu et al. [41]. Each of the LT muscle samples were frozen in liquid nitrogen and embedded in Tissue-Tek (Sakura Finetek Europe, Zoeterwoude, The Netherlands). Subsequently, serial cross-sections (10 μm thick) were cut with a cryostat at −20 °C and placed on poly-l-lysine-coated glass slides (Sigma-Aldrich, St. Louis, MO, USA), and they were stained for 30 s in haematoxylin. Between 100 and 150 cross-sections of fibre, randomly selected, were analysed per sample of LT from different production systems and ageing treatments. To determine the mean transverse of the muscular fibre (μm^2^) by computerised image analysis, the Nis-Elements 3.10 software with a magnification of 100× was used. Structural elements were measured in a fibre bundle area, and more than 200 sections of samples were analysed per sample of LT from different ageing treatments and production systems.

### 2.5. Statistical Analysis

A two-way ANOVA test was carried out to determine the statistical significance for the 3 × 4 factorial design. The model included the fixed effects of the production system (OG, OC, and CC) and ageing time (D_0_, D_7_, D_14_, and D_21_) and their interactions on pH, water losses, instrumental textural properties, and histological parameters of LT muscle samples. Since there were significant differences in the slaughter weight among production systems, the effect of slaughter weight was used as a covariate. Slaughter weight was not included in the final model, because it did not have a significant influence on the parameters under study. The HSD Tukey’s test was used to compare means, with significance being set at *p* ≤ 0.05. Mean values and standard errors of the means (SEM) for all studied variables were reported. The relationship between the histological analysis variables of muscle fibre (sarcomere length and cross-sectional area of fibre) and texture parameters (Warner-Bratzler shear force and Texture Profile Analysis) were calculated by Pearson correlation coefficient (*r*).

## 3. Results and Discussion

### 3.1. pH and Water Losses (Drip and Cooking Losses)

The pH values of the LT muscle are shown in Table 1. An interaction between production system and ageing time was observed (*p* ≤ 0.05) on pH. Consequently, the evolution of the pH throughout the ageing period was determined by the pH_24_ of the meat in each production system. Organic beef from the OG and OC production systems showed a lower pH than meat from the CC system. These values were below 6.0, which is in the normal range for beef [42], and therefore, it is without consequences from the point of view of meat quality. Our results are in accordance with those observed by Avilés et al. [43] for the Retinta breed. Regarding the production system, our findings are in line with other authors [25,44] who observed a lower pH value in beef from extensive production than in confined cattle. However, since the post-mortem decline in pH is related to muscle glycogen reserves, for confined cattle, which are accustomed to handling and contact with people, a lower pH due to a lower consumption of muscle glycogen was expected because of pre-slaughter stress [25]. Thus, any factor that increases the glycogen depletion rate leads to a decrease in substrate for post-mortem anaerobic glycolysis [45,46] and the consequent higher pH. The pH_24_ determines the quality characteristics of the meat. Specifically, Jeleníková et al. [47] concluded that there was a curvilinear relationship between the tenderness of the *Longissimus lumborum et thoracis* muscle and the final pH. Concerning ageing time, a significant effect (*p* ≤ 0.05) on the pH values of the LT muscle was observed, which was also reported by Franco et al. [48].

Table 1 shows the results of the effect of the production systems and ageing period on water losses (DL and CL). The water losses were affected by the significant interaction between the production system and ageing time factors (*p* ≤ 0.001). These findings indicated that the effect of ageing time should be assessed within each production system.

Differences between meat produced under organic production systems (OG and OC) and meat produced under CC systems were observed, which showed the highest degree of DL in CC. Concerning the ageing time, the highest water loss was observed between T_14_ and T_21_ (*p* ≤ 0.05). Drip loss is as assessment of the loss of fluid from beef cuts due to the shrinkage of muscle proteins (actin and myosin) in the form of drip. Several factors influence it, such as the breed [49,50,51] and the production system [44,52]. The results of water loss measured as CL (Table 1) showed differences due to the production system effect. The findings showed the opposite in relation to DL values, as Olsson et al. [53] reported for organic pork. Thus, the meat from organic production systems (OG and OC) showed higher CL than the meat from CC. Although the water content in meat plays an important role in the perception of juiciness and tenderness by consumers, to our knowledge, the scientific literature is scarce about assessing water losses between organic versus conventional beef. Miotello et al. [14] observed no differences on cooking water loss between organic and conventional beef. Ribas-Agustí et al. [12] observed higher water content measured by using a near-infrared meat analyser, but controversially, they found no difference with conventional beef in a meta-analysis. On the other hand, Walshe et al. [13] evaluated the total water content (moisture content) and did not observe differences due to the organic versus conventional production system. The ageing process affected cooking water loss; thus, the highest CL values were observed after 7 days of ageing (T_7_) and the lowest value in T_21_. However, Straadt et al. [54] reported that cooking water loss increased up to 4 days after slaughter, and Palka [55] observed that the cooking losses were lower in meat when the ageing period increased.

While drip loss occurs due to the degradation of the cytoskeletal protein during ageing [56] and to the formation of drip channels due to the disappearance of the link between myofibrils, which is caused by enzymatic action [57], cooking water loss is due to the denaturation of muscle proteins caused by the effect of heat, which affects their structural characteristics [57]. Several studies indicated the relationship between the drop in pH and lower WHC due to the denaturation of proteins [57], thereby facilitating the formation of drip channels. However, in light of our findings, the lower water losses (drip loss) in meat from organic systems could be also explained by other factors such as the structural and metabolic muscular changes [25,58] produced by the physical exercise due to the greater availability of space in these systems (OG and OC) and a greater consumption of mainly α-tocopherol from the grass in their diet (results not shown), which has also been reported by other authors [12,59]. Such factors help avoid lipid oxidation and stabilise the cell membranes, thus delaying the loss of water [59].

### 3.2. Warner-Bratzler Shear Force Test (WB)

Table 1 shows the effect of the production system on instrumental texture parameters, as measured by the Warner-Bratzler test on cooked meat. The interactions between production systems and ageing time were significant in all studied WB parameters (*p* ≤ 0.001); consequently, the interpretation of the main effects is complex, which suggested that they should be assessed within each production system. Although some authors [9,11] observed no differences on the shear force due to organic or non-organic production systems, our results indicated the importance of this effect. According to Vestergaard et al. [20], the higher level of physical activity on grazing, compared with the limited activity in intensive condition, is the main reason for the highest WB parameter values, and therefore a lower tenderness. Physical exercise is associated with the extensive production system [19], which involves a structural and biochemical change in the muscle fibres [60]. These reasons could explain our results obtained for OG meat in shear force parameters, which were probably due to the availability of space to do physical exercise alongside the grass-feeding. Some authors observed that the muscles undergo a series of physical and biochemical changes due to exercise-linked grass-feeding [18,19,61]; Jurie et al. [60] observed an increase in oxidative muscle fibres, which was mainly related to the grass-feeding system affecting quality aspects such as colour and texture.

However, other factors such as diet could be taken into account, especially when it contains a high level of fat and high marbling scores in the meat [62]. Although Giaretta et al. [63] observed a positive correlation between meat tenderness and fat content, in particular to intramuscular fat [64,65], other authors reported that the diet is probably not the major cause of tenderness differences [43]. These authors reported that the fatness scores of Retinta carcass showed lower subcutaneous fat thickness than other rustic breeds, and they had low intramuscular fat values (<2%) under different types of feeding, which could be a reason for thinking about the importance of exercise in the above diet.

Considering that there is a relationship between shear force and hardness as sensory attributes [66], our results, instrumentally obtained, are consistent with the assessment sensorial of Retinta organic beef carried out by consumers [67], in which they rated meat from organic grass-fed animals (OG) as the toughest.

The ageing period had an effect on WB parameters (Table 1). All the parameters (shear force, shear firmness, and total work) decreased over ageing time (*p* ≤ 0.001), as observed by other authors [19,51]. At T_0_, the highest WB parameters values were observed, and in both shear force and total work, values reduced from T_0_ to T_14_, although after 14 days of ageing, no changes were observed in these parameters. In the present study, shear force values at 14 days of ageing were higher than those reported by other authors [51,68] for conventional meat from the Retinta breed.

**Figure 1:**
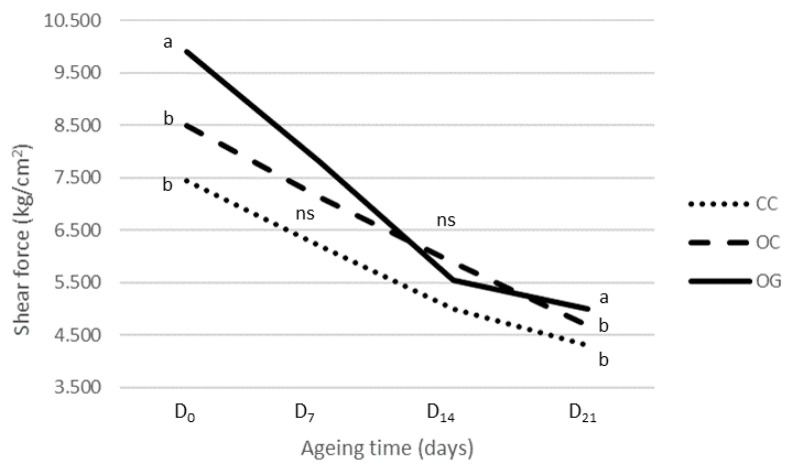
Evolution of the shear force values of *Longissimus thoracis* muscle from calves of the Retinta breed throughout 21 post-mortem days of ageing in organic grazing (OG), organic concentrate (OC), and conventional concentrate (CC) systems. Values with the same letters (a, b) indicate homogeneous subsets between productions systems for *p* = 0.05 according to Tukey’s HSD test; ns = not significant.

Figure 1 shows the different tenderisation patterns among OG, OC, and CC meats throughout the ageing process. Although at the end of the ageing period (T_21_) the meat in the three production systems showed no differences in shear force values, the reduction in shear force at 7 days of ageing was 42.65% for OG, 36.22% for OC, and 39.83% for CC, and after the first week, no significant differences among the production systems were shown; therefore, differences in the tenderisation pattern were identified. Thus, while the tenderisation speed of OC and CC meat was similar, OG meat revealed a totally different pattern of tenderisation. Initially, it was the toughest, and it had a quicker tenderisation during the first days of ageing. This finding leads us to consider that the differences found are mainly related to the extensiveness of the OG production system, in which greater exercise affects the myofibrillar structure and characteristics of muscle, causing an increase in meat tenderness after a long ageing period [19,60].

### 3.3. Texture Profile Analysis (TPA)

Table 2 shows the results of effect of the production systems and ageing time on texture profile analysis at a compression ratio of 20% (TPA20). The compression parameters obtained with TPA have been used by many authors to effectively evaluate the textural properties of meat products in both raw and cooked meat [36,69,70]. The differences in the textural parameters of TPA20 are due to the behaviour of the myofibrillar structures without the intervention of the connective tissue [71].

**Figure 2:**
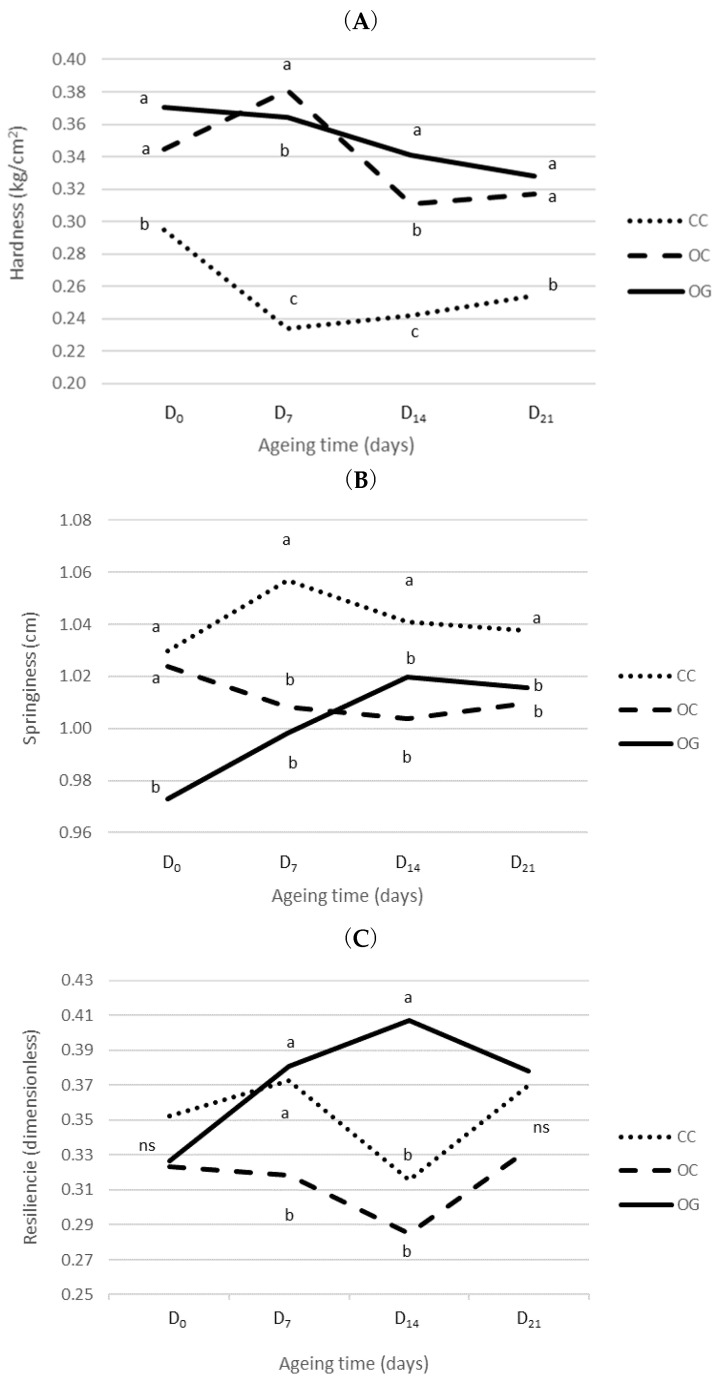
Interaction effects between production system and ageing time on TPA20 of *Longissimus thoracis* muscle from calves of the Retinta breed throughout 21 post-mortem days of ageing on Hardness (**A**), Springiness (**B**) and Resilence (**C**). Values with the same letters (a, b, c) indicate homogeneous subsets for *p* = 0.05 between productions systems according to Tukey’s HSD test; ns = not significant; OG: organic grazing; OC: organic concentrate; CC: conventional concentrate.

Figure 2 showed that the production system had a significant interaction with ageing time for hardness (*p* ≤ 0.001), springiness (*p* ≤ 0.01), and resilience (*p* ≤ 0.05), and the beef from each production system had different behaviour depending on the studied ageing time. Both the hardness and springiness parameters showed similar behaviours in organic meat (OG and OC), which were differentiated from conventional meat (CC).

Regarding hardness, the OG meat showed the highest values, the OC meat showed intermediate values, while the lowest values were found in the CC meat. The CC meat reached the lowest hardness value after 7 days of ageing, followed by the OC meat, which reached the lower value after 14 days, and finally the OG meat, which remained constant. On the other hand, the production system had a major weight on the interaction of the springiness, because ageing time showed no significant effect on this parameter. In the case of resilience, the highest value in OG meat was observed, and for both OC and CC, the lowest values were reached at 14 days of ageing. However, the chewiness and cohesiveness did not show significant interactions, and the main effects could be analysed separately. In both parameters, the production system effect results were significantly different (*p* ≤ 0.001). The organic systems (OG and OC) showed the highest values of chewiness, and the OG meat showed the highest cohesiveness.

Thus, according to the definition of the parameters of Icier et al. [72], the organic beef (OG and OC) showed higher chewiness (length of time required to chew a sample to a consistency suitable for swallowing) and a lower springiness value (degree to which a product returns to its original shape once it has been compressed) than CC beef. In particular and in line with the one previously observed in shear force, the OG meat presented higher hardness (force necessary to attain a given deformation, maximum force) and cohesiveness values (strength of the internal bonds making up the body of the product).

Despite the difficulty of evaluating each factor linked to the production system individually (breed, gender, diet, available space, or exercise), due to the interaction among them, and in particular under extensive conditions, where grazing involves higher exercise, some studies suggest that the diet of the animals is not the most important factor in the tenderness of the meat [43]. Several studies had reported the exercise factor as determinant of meat texture characteristics. Thus, Aalhus and Price [73] concluded that exercise is a factor that can influence the type, size, and composition of muscle fibre and, therefore, this affects meat texture characteristics. The same finding was also reported by Jurie et al. [60], even when the space available was smaller. In light of our results, where each studied group had different available space, and according to the previous authors, we could highlight the exercise as a differentiating factor involved in the animals grazing.

Regarding the ageing time effect, it was noted that ageing time had a significant effect on chewiness (*p* ≤ 0.05) parameters. As expected, at D_0_, the hardness parameter showed the highest value, and the lowest value was observed in the chewiness parameter.

The effect of ageing time on texture characteristics is described as an effect that is more important than breed [22] and other factors.

### 3.4. Muscle Fibre Characteristics

Table 3 shows the results obtained for the histological parameters of LT muscle fibres in Retinta calves. These results are in agreement with those found by other authors for beef (2.2 μm) [74]. The relationship between the characteristics of the muscle fibres and the texture of the meat has been extensively studied, both the relationship with the length of the sarcomere [75] and with the area of the muscle fibre [76,77,78].

The production system had a significant effect on sarcomere length (*p* ≤ 0.001) and muscle fibre cross-sectional area (*p* ≤ 0.001). OG meat muscle fibre was found to have a shorter sarcomere and larger fibre cross-sectional area than OC and CC. On the other hand, OC meat showed intermediate values on cross-sectional area, while the lowest values were found in the CC meat. Muscle fibre structures can change due to several factors such as age or exercise [79,80], which supports our hypothesis that extensive systems have a great influence on texture properties. As other authors indicated [20,60], the extensive production system—due mainly to physical exercise—affects the histological characteristics of muscle fibres.

The histological parameters analysed (sarcomere length and cross-sectional area of fibres) showed significant differences throughout ageing time (Table 3). An increase in sarcomere length during ageing time (*p* ≤ 0.001) was observed. This result is according with the findings of Viera and Fernández [81], who reported that the length of sarcomeres increases with ageing time and is closely related to shear force, which is in line with the observations of Ertbjerg and Puolanne [82]. In our study, the lowest sarcomere length value was observed at T_0_, and the highest was observed at T_21_. The greatest elongation of the sarcomere between two ageing times was observed from T_7_ to T_14_, and this result is consistent with the T_14_ instrumental measurements of shear force, although Starkey et al. [83] suggested that there is still a variation in shear force that cannot be completely explained by histological parameters such as sarcomere length, even though it is moderately correlated with shear force. Concerning the cross-section area, during 21 days of ageing, a continuous reduction until around 20% of the fibre cross-sectional area was noted, with the greatest decrease occurring between days T_0_ and T_7_.

### 3.5. Relationship between Texture Parameters and Muscle Fibre Characteristics

Table 4 shows the correlation coefficients between histological and instrumental texture variables. Our data confirm a negative correlation between cross-sectional area and sarcomere length (−0.284; *p* ≤ 0.01). A negative correlation between sarcomere length (−0.355; *p* ≤ 0.01) and the shear force was also observed. These results agree with those reported by Janz et al. [84], which established a correspondence between a longer sarcomere length and a significantly lower shear force value. Rhee et al. [75] also found that the overall sarcomere length was significantly correlated to tenderness, and Pen et al. [85] reported that sarcomere length plays an important role in meat tenderness, particularly in the first few days of ageing until proteolysis exerts a greater influence than sarcomere length on the final texture of the meat, since the ageing of beef is considered the most important factor myofibrillar tenderisation [22]. On the other hand, Żochowska et al. [77] and Guillemin et al. [78] informed that meat with higher fibre diameters had greater shear strength, although ours findings showed no correlation (*p* > 0.05).

Sarcomere length was negatively correlated with some TPA20 parameters as hardness (−0.444; *p* ≤ 0.01), resilience (−0.180; *p* ≤ 0.05), and cohesiveness (−0.415; *p* ≤ 0.01), and it was correlated positively with chewiness (0.372; *p* ≤ 0.01) and springiness (0.362; *p* ≤ 0.01). On the other hand, cross-section area values showed positive correlations on hardness (0.261; *p* ≤ 0.01) and cohesiveness (0.254; *p* ≤ 0.01), whilst the correlation was negative with springiness (−0.215; *p* ≤ 0.05).

A lack of correlation between Warner-Bratzler and compression test (TPA) parameters is usually found [68], as noted in this study. In this sense, our results only indicated a positive correlation between shear force and chewiness (0.393; *p* ≤ 0.01). Caine et al. [86] observed a similar value for this correlation, and they suggested that both instrumental methods (Warner-Bratzler and TPA tests) could be useful in assessing meat hardness. Thus, TPA could be a valid method for the evaluation of meat texture characteristics [36].

## 4. Conclusions

OG production systems resulted in meat with higher instrumentally measured textural values (Warner-Bratzler and TPA at 20% compression). Overall, the meat was harder, with less springiness, more resilient, and more cohesive than OC and CC meat samples. In spite of these characteristics, the OG meat pattern of tenderness, i.e., the fast decrease of shear force values throughout its ageing time, was greater in the first days of ageing when compared to the ones from the other systems studied. After 14 days, meat tenderness is similar in the three systems, and it can now be established that 14 days is the ageing time needed for the Retinta breed meat to reach its maximum value of tenderness. As a consequence, extending the ageing time does not improve meat tenderness; however, it could result in an increase of production costs. Furthermore, and in line with instrumental texture results, our findings show a direct association between muscle fibre characteristics, which showed changes throughout the ageing period, and meat tenderness from the different production systems. Thus, the sarcomere’s length was directly proportional to those parameters related to tenderness (shear force or hardness), while the cross-sectional area of muscle fibres was inversely proportional to them. However, more biochemically or histochemically oriented research is needed to better understand the effect of differentiating factors of the production systems (available space or exercise) on textural beef characteristics.

Given our results, Retinta organic meat is a more suitable choice for consumers who, compared to a conventional diet, perceive organic food as more valuable, since tenderness has been associated to meat repurchase consumer behaviour. Thus, this positive consumer experience of organic Retinta meat would contribute to consumer loyalty and improve the development of organic meat production.

## Figures and Tables

**Table 1:** Effect of the production systems (organic grazing, OG; organic concentrate, OC; and conventional concentrate, CC) and ageing time (T_0_, T_7_, T_14_, T_21_: 0, 7, 14, and 21 days, respectively) on pH, water-holding capacity, and textural instrumental parameters of *Longissimus thoracis* from calves of the Retinta breed.

	Production System (PS)			Ageing Time (A)					Effects		
	OG	OC	CC	T_0_	T_7_	T_14_	T_21_	SEM	PS	A	PS*A
pH	5.55 ^b^	5.54 ^b^	5.60 ^a^	5.52 ^b^	5.56 ^a,b^	5.60 ^a,b^	5.63 ^a^	0.017	*	*	*
Drip Loss (g/100 g)	5.42 ^b^	4.44 ^b^	6.80 ^a^	-	3.04 ^c^	4.28 ^b^	6.48 ^a^	0.197	***	***	***
Cooking Loss (g/100 g)	24.48 ^a^	25.36 ^a^	22.25 ^b^	25.61 ^b^	27.51 ^a^	24.97 ^b,c^	23.64 ^c^	0.217	***	***	***
Warner-Bratzler shear force test											
WB-Shear Force (kg/cm^2^)	6.79 ^a^	5.21 ^b^	5.08 ^b^	8.66 ^a^	6.67 ^b^	5. 04 ^c^	4.32 ^c^	0.171	***	***	***
WB-Shear Firmness (kg/s)	11.23 ^a^	8.15 ^b^	8.76 ^a,b^	13.07 ^a^	9.82 ^b^	8.64 ^b,c^	7.37 ^c^	0.25	***	***	***
WB-Total Work (kg*s)	1.30 ^a^	1.15 ^b^	1.07 ^b^	1.71 ^a^	1.27 ^b^	1.02 ^c^	0.90 ^c^	0.03	***	***	***

* *p* ≤ 0.05; *** *p* ≤ 0.001; PS: production systems; A: ageing time; PS*A interaction between A and PS; SEM.: standard error of the mean. Values with the same letters (a, b, c) indicate homogeneous subsets for *p* = 0.05 according to Tukey’s HSD test; OG: organic grazing; OC: organic concentrate; CC: conventional concentrate.

**Table 2:** Effect of the production systems and ageing time (T_0_, T_7_, T_14_, and T_21_: 0, 7, 14, and 21 days, respectively) on texture profile analysis 20% (TPA20) of *Longissimus thoracis* from calves of the Retinta breed.

	Production System (PS)			Ageing Time (A)					Effects		
	OG	OC	CC	T_0_	T_7_	T_14_	T_21_	SEM	PS	A	PS*A
Hardness (kg/cm^2^)	0.51 ^a^	0.33 ^b^	0.25 ^c^	0.39 ^a^	0.41 ^a^	0.35 ^b^	0.36 ^b^	0.010	***	***	***
Springiness (cm)	1.00 ^b^	1.01 ^b^	1.04 ^a^	1.00	1.02	1.01	1.02	0.003	***	ns	**
Resilience (non-dimensional)	0.37 ^a^	0.15 ^b^	0.35 ^b^	0.35	0.36	0.35	0.34	0.003	***	ns	*
Chewiness (kg·m·s^−2^)	0.34 ^a^	0.32 ^a^	0.28 ^b^	0.30 ^b^	0.31 ^a^	0.27 ^a,b^	0.24 ^a,b^	0.005	***	*	ns
Cohesiveness (non-dimensional)	0.65 ^a^	0.62 ^b^	0.62 ^b^	0.64	0.64	0.63	0.63	0.003	***	ns	ns

ns: *p* > 0.05; ** *p* ≤ 0.01; *** *p* ≤ 0.001; A: ageing time; PS: production systems; PS*A: interaction between A and PS; SEM.: standard error of the mean. Values with the same letters (a, b, c, d) indicate homogeneous subsets for *p* = 0.05 according to Tukey’s HSD test. OG: organic grazing; OC: organic concentrate; CC: conventional concentrate.

**Table 3:** Effect of the production systems (organic grazing, OG; organic concentrate, OC and conventional concentrate, CC) and ageing time (T_0_, T_7_, T_14_, T_21_: 0, 7, 14, and 21 days, respectively) on histological parameters of *Longissimus thoracis* from calves of the Retinta breed.

	Production System (PS)			Ageing Time (A)					Effects		
	OG	OC	CC	T_0_	T_7_	T_14_	T_21_	SEM	PS	A	PS*A
Sarcomere length (μm)	2.31 ^b^	2.89 ^a^	2.98 ^a^	1.92 ^d^	2.14 ^c^	3.02 ^b^	3.40 ^a^	0.026	***	***	***
Cross-section Area (μm^2^)	1291.24 ^a^	1177.75 ^b^	921.61 ^c^	1217.27 ^a^	1108.32 ^b^	1035.07 ^b,c^	978.33 ^c^	13.525	***	***	***

*** *p* ≤ 0.001; A: ageing time; PS: production systems; PS*A: interaction between A and PS; SEM. standard error of the mean. Values with the same letters (a, b, c, d) indicate homogeneous subsets for *p* = 0.05 according to Tukey’s HSD test. OG: organic grazing; OC: organic concentrate; CC: conventional concentrate.

**Table 4:** Pearson’s correlation coefficient (*r*) between histological and texture parameters of *Longissimus thoracis* from calves of the Retinta breed.

	Sarcomere	Area	Shear F.	Hard20	Spri20	Chew20	Res20	Coh20
Sarcomere	1							
Area	−0.284 **	1						
Shear F.	−0.355 **	−0.080	1					
Hard20	−0.444 **	0.261 **	−0.192	1				
Spri20	0.372 **	−0.215 *	0.152	−0.956 **	1			
Chew20	0.362 **	−0.162	0.393 **	−0.382 **	0.475 **	1		
Res20	−0.183 *	0.110	−0.062	0.847 **	-0.866 **	−0.163 *	1	
Coh20	−0.415 **	0.254 **	−0.264	0.985 **	-0.947 **	−0.394 **	0.872 **	1

* *p* ≤ 0.05; ** *p* ≤ 0.01; Sarcomere: sarcomere length; Area: cross-section area; Shear F: shear force; compression test TPA at 20% deformation: Hard20 (hardness); Spri20 (springiness); Chew20 (chewiness); Res20 (resilience); Coh20 (cohesiveness).
